# Molecular characterization of marine bacterial isolates of Visakhapatnam coast—efficacy in dye decolorization and bioremediation of cadmium

**DOI:** 10.1186/s43141-021-00189-0

**Published:** 2021-06-16

**Authors:** Teja Mandragutti, Muni Kumar Dokka, Bindiya Panchagnula, Sudhakar Godi

**Affiliations:** 1 Department of Biotechnology, Andhra University, Visakhapatnam, 530 003 India; 2 Department of Biochemistry, Andhra University, Visakhapatnam, 530 003 India; 3 Department of Human Genetics, Andhra University, Visakhapatnam, 530 003 India

**Keywords:** *Bacillus subtilis*, *Pseudomonas resinovorans*, 16S rRNA, Phylogenetic analysis, Bioremediation

## Abstract

**Background:**

Microbial community is one of the diversified communities of the marine environment. Studies have shown that microorganisms isolated from the marine environment are metabolically active and have adapted to life in the ocean. The marine microorganisms use various survival strategies to combat heavy metal stress and decolorization of various textile dyes, thus playing an important role in the bioremediation of cadmium and degradation of textile dyes. The present study deals with the isolation and 16S rRNA molecular characterization of M3 and M8 bacterial strains isolated from marine water samples collected from Visakhapatnam harbor. M3 and M8 isolates were also checked for their efficacy in the removal of cadmium and decolorization of various textile dyes from the environment.

**Results:**

The water sample was subjected to tube dilution method to isolate bacterial strains, and ten different isolates were screened. The biochemical tests were performed for the isolates to prove their validity and 16S rRNA molecular sequencing and phylogenetic analysis for species identification. Out of interest, two bacterial strains, namely, M3 and M8 were subjected to 16S rRNA molecular sequencing and phylogenetic analysis and were identified as *Bacillus subtilis* and *Pseudomonas resinovorans*. The two bacterial strains showed promising dye degradation property when checked with nine different textile dyes of wavelength ranging from 400 to 600 nm and removal of cadmium from the growth medium.

**Conclusion:**

The present study demonstrates the isolates M3 and M8 to be potential strains having dye decolorization and bioremediation of cadmium applications.

**Supplementary Information:**

The online version contains supplementary material available at 10.1186/s43141-021-00189-0.

## Background

The marine environment covers approximately 70% of earth surface and contains vast biological diversity which accounts for more than 90% of the whole biosphere and offers a great source of novel compounds [1]. The marine environment is a prolific resource for the isolation of less exploited microorganisms and represents a largely untapped source for the isolation of new microorganisms [2]. Thus, marine ecosystems and coastal regions are particularly promising because of the rightly adapted species in the harsh environment. Bacteria from marine environment grow in extreme environmental conditions such as high pressure, low temperature, high salinity, and depletion of micronutrients [3]. Hence, the exploration of marine microorganisms from marine environment has led to the discovery of hundreds of microbes with biologically active compounds and versatile properties like bioremediation, biodegradation of textile dyes, and bioleaching. Marine microorganisms have become significant source of novel microbial products exhibiting antibacterial, anticancer, anti-viral, anti-coagulant, anti-inflammatory, antioxidant, and cardioprotective properties [4]. Varied types of halophilic and halotolerant microbes have been isolated from broad range of aquatic environment [5]. Species-specific variability regions of 16S rRNA gene sequence analysis is the most supportive for the classification of phylogenetic uniqueness in conformity to the phenotypic profiles for the identification of microbes [6].

The textile industry plays a crucial role in the global economy as well as in the daily life and simultaneously becoming one of the main sources of environmental pollution in the world in terms of quality and quantity [7]. The textile industry consumes a larger volume of water in which roughly 90% appear as wastewater [8]. Textile wastewater contains various types of dyes as major pollutants which not only is recalcitrant but also imparts intense color to the waste effluent [7]. Inappropriate dumping of textile wastewater causes serious environmental hazards that affect the aquatic organisms badly [9]. Inappropriate effluent disposal in aqueous ecosystems leads to reduction of sunlight penetration which in turn diminishes photosynthetic activity, resulting in acute toxic effects on the aquatic flora and fauna and dissolved oxygen concentration [10]. Increased biological oxygen demand (BOD) and chemical oxygen demand (COD) values of the dye effluent make it complicated to degrade and produce a toxic environment for aquatic biodiversity [11]. So, their treatment before releasing to the environment is so essential to minimize water pollution. Among different treatment processes, bio-treatment has regarded the most efficient way in comparison with conventional physicochemical processes in the degradation of textile dyes [12]. Microbial decolorization and degradation is an environmental-friendly and cost-competitive alternative to chemical decomposition processes [13]. The ability of microorganisms to carry out dye decolorization has recently received much attention as microbial decolorization of dyes is a cost-effective method for removing them from the environment [14]. Remediation of dyeing industry effluent by using microorganisms has proved to be the best solution, since numerous bacterial species including *Bacillus*, *Pseudomonas*, *Enterobacter*, *Halobacter*, and *Aeromona*s have been reported to exhibit incredible capability to decolorize and detoxify a wide range of dyes [15, 16].

Bioremediation is an innovative and promising technology available for removal of heavy metals and recovery of the heavy metals in polluted water and lands. Since microorganisms have developed various strategies for their survival in heavy metal-polluted habitats, these organisms are known to develop and adopt different detoxifying mechanisms such as biosorption, bioaccumulation, biotransformation, and biomineralization, which can be exploited for bioremediation either ex situ or in situ [15, 17]. Bioremediation has become one of the promising in situ technologies for the clean-up of environmental pollutants using microorganisms. Environmental pollution with heavy metals is increasing day by day due to urbanization and industrialization [18] and became a major global concern because of its toxicity and threat to human life and environment. Nowadays, the bioaccumulation of heavy metals in environment is a major warning to human life [19, 20].

Metals like copper, iron, manganese, and zinc are essential for life processes whereas others like cadmium, lead, nickel, and mercury have no physiological function but often results in harmful disorders at a higher concentration [21]. Many heavy metals are even non-degradable in nature and hence once released into the environment remain in circulation. Heavy metals containing industrial effluents lead to health hazards to plants, animals, aquatic life, and humans and thus increasing pressures on the flora and fauna [22]. Lead and cadmium which are major contaminants found in the environment are extremely poisonous to humans, animals, plants, and microbes which can damage cell membranes, alter functions of enzymes, and damage the structure of DNA. Microbes have evolved mechanisms such as active efflux or sequestration with proteins or insoluble compounds through which they may resist, detoxify, or metabolize these heavy metals. It is important to note that bioremediation technologies based on microbes are economically viable, cost-effective, and environment friendly.

Cadmium (Cd) is identified as a major pollutant, non-essential metal, and is harmful to living organisms at relatively low concentrations, i.e., about 0.001–0.1 mg/L [23]. It is well known that cadmium is not involved directly in any known biological processes as a co-factor or activator, but is known to inhibit several enzyme activities, involved in the inhibition of DNA-mediated transformation in microorganisms and interference in the symbiosis between microbes and plants, as well as involved in plant predisposition to fungal invasion [24].

Hence, by considering the scope of marine bacteria and the less exploited nature of marine microorganisms, the present study has been taken up to isolate and characterize bacteria from marine water collected from Visakhapatnam harbor, and an attempt has been made to check the efficiency of M3 and M8 strains for their decolorization and bioremediation properties.

## Methods

### Sample collection

Marine water sample was collected in sterile bottles from Visakhapatnam harbor (17.6958° N, 83.3025° E), Andhra Pradesh, and brought to laboratory and stored at 4°C until used for the isolation of bacteria.

### Isolation of bacteria

Quantitative estimation of the viable bacteria was done by serial tube dilution method, plating, and colony count. The bacteria were isolated by spread plate method on nutrient agar medium, incubated at 37°C for 24 h to obtain colonies. The individual colonies were picked upon the basis of their macroscopic characters such as size, shape, surface appearance, texture, and color. These colonies were subjected to repeated streaking on nutrient agar plates/slants. The so obtained marine bacterial isolates were stored at 4°C for further studies.

### Morphological characterization of isolated bacteria

Colony and cell morphology based on their color, shape, margin, elevation, surface, and arrangement of bacteria were studied.

### Gram staining

The standard Gram staining procedure was followed for the morphological characterization of isolates [25].

### Biochemical characterization of isolated bacteria

For the taxonomic identification, different biochemical tests were performed for all the ten isolates to check their metabolic activities following standard protocols [26].

### Identification of isolated bacteria by 16S rRNA gene sequencing

The isolates M3 and M8 were subjected to molecular characterization by16S rRNA sequencing. The DNA of the isolates was extracted using a standard protocol followed by Rohini et al. [27]. Confirmation of DNA was done using agarose gel electrophoresis on 1% agarose gels. Fragment of 16S rRNA gene was amplified by 27F (5-GAGTTTGATCCTGGCTCA-3) and 1492 (R-TACGGYTACCTTGTTACGACTT) universal primers. PCR reaction was performed with the conditions such as initial denaturation at 94°C for 2 min, 35 amplification cycles at 94°C for 45 s, 55°C for 60 s, 72°C for 60 s, and a final extension at 72°C for 10 min. The PCR amplicon was purified to remove contaminants. Forward and reverse DNA sequencing reaction of PCR amplicon was carried out with forward primer and reverse primers using BDT v3.1 Cycle sequencing kit on ABI 3730xl Genetic Analyzer. Consensus sequence of 16S rRNA gene was generated from forward and reverse sequence data using an aligner software.

### Construction of phylogenetic tree

The 16S rRNA gene sequence of the two isolates was used to carry out BLAST with the database of NCBI Genbank. Based on maximum identity score, first ten sequences were selected and aligned using multiple alignment software program Clustal W. Distance matrix was generated, and the phylogenetic tree was constructed using MEGA 7.

### Applications of M3 and M8 bacterial strains

#### Decolorization of dye compounds

The two isolates were grown in 100 ml minimal salt medium (MSM) containing selected dye compounds in a conical flask at 25°C and continuous shaking at 100 rpm. The composition of MSM is yeast extract (1g/L), (NH_4_)_2_SO_4_ (2.5g/L), KH_2_PO_4_(13.3g/L), Na_2_HPO_4_(21.6 g/L), and glucose (1.25g/L). The experiment was carried out using 50 mg of each dye (Trypan blue, Methyl red, Neutral red, Congo red, Bromophenol blue, Coomassie brilliant blue, and Gentian violet) in 1 L of MSM separately. The isolates were inoculated in the sterile liquid minimal media in conical flasks and incubated at room temperature using shake-flask method. Samples were withdrawn at the intervals of 12 h, 24 h, 48 h, 72 h, and 96h during the incubation and checked for decolorization of dye compounds using spectrophotometric analysis. A small aliquot of the media was extracted in sterile conditions. The aliquot was subjected to centrifugation at 10,000 rpm for 5 min. The supernatant was collected, and the absorbance was noted using the sterile uninoculated media without dye as a blank. The absorbance for different dyes was noted at their respective wavelengths.

The decolorization of the media indicates the degradation of the dye by the bacteria.

The efficiency of degradation of the dye can be calculated using the following formula:

$$ \%\kern0.5em \mathrm{Degradation}=\frac{\left({\mathrm{A}}_0\hbox{-} {\mathrm{A}}_{\mathrm{t}}\right)}{{\mathrm{A}}_0}\mathrm{X}\kern0.5em 100 $$

where A_0_ is the initial absorbance of the media and A_t_ is the absorbance of the media at the interval of “t” time.

#### Cadmium removal capacity

The isolated strains, M3 and M8, were checked for their cadmium removal capacity from the growth medium, Luria Bertani broth containing 100 휇g/ml of the cadmium. During the growth period, 1 ml of each bacterial culture sample was removed into Eppendorf tubes after every 4 h until 24 h and centrifuged at 6000 rpm for 15min. An extra sample without the addition of bacterial culture was taken as control. The supernatants were collected and stored at 4°C for the cadmium analysis. The cadmium concentrations in the supernatants were analyzed using Atomic Absorption Spectrometer (Agilent Technologies Pvt, Ltd., Australia) at 228.8 nm with a cadmium hollow cathode lamp. The percentage of cadmium concentration in the growth medium was calculated at every 4 h intervals. The optimal density of each sample was also measured at 600 nm to compare the growth rate of bacteria with the cadmium removal capacity [28].

## Results

### Isolation of bacteria and morphological characterization of bacteria

A total of ten different bacterial colonies were isolated from marine water sample on the basis of unique colonial characteristics on nutrient agar medium. Morphological and colony characteristics for the isolated bacteria were observed and tabulated (Table 1). All the ten bacterial isolates exhibited different morphological and colonial characteristics.

**Table 1:** Morphological characterization of isolated bacterial strains

S.NO	Morphology & colony characteristics	M1	M2	M3	M4	M5	M6	M7	M8	M9	M10
1.	Gram reaction	+ve	-ve	+ve	+ve	-ve	+ve	+ve	-ve	-ve	+ve
2.	Shape	Rod	Cocci	Rod	Cocci	Cocci	Rod	Cocci	Cocci	Rod	Rod
3.	Colony size	Large	Large	Medium	Medium	Medium	Small	Small	Small	Large	Large
4.	Colony shape	Filamentous	Circular	Circular	Circular	Circular	Irregular	Circular	Circular	Filamentous	Circular
5.	Colony elevation	Flat	Umbonate	Flat	Crateriform	Convex	Flat	Convex	Convex	Flat	Convex
6.	Surface	Dry	Moist	Moist	Moist	Moist	Dry	Moist	Moist	Powder	Moist
7.	Opacity	Opaque	Transparent	Opaque	Transparent	Transparent	Opaque	Transparent	Transparent	Opaque	Transparent
8.	Pigmentation	No	Yes	No	Yes	No	No	Yes	Yes	No	No

*+ve* gram positive, *-ve* gram negative

### Biochemical characterization of bacterial isolates

**Fig. 1:**
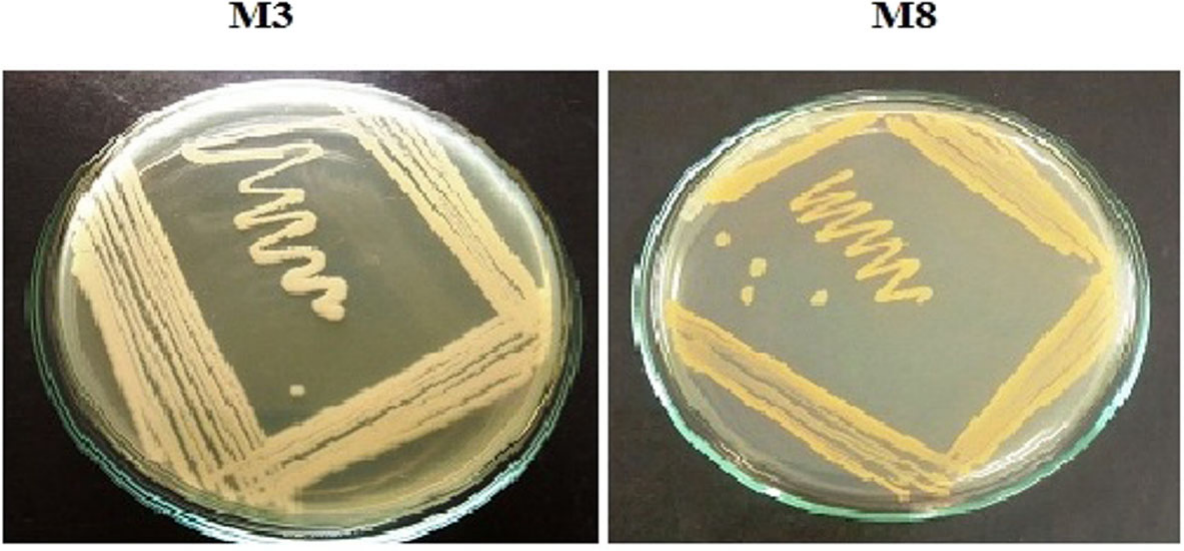
M3*—Bacillus subtilis*, M8—Pseudomonas resinovorans

For taxonomic identification, the isolates were subjected to a series of biochemical tests which includes mannitol salt agar, methyl red test, Voges-Proskauer test, Indole test, citrate utilization test, oxidase test, urease test, gelatine liquefaction test, catalase activity, H_2_S production, motility, starch hydrolysis, hemolysis, and carbohydrate fermentation tests, and the results are shown in Table 2. The isolate differed in acid production, H_2_S production, methyl red test, Voges-Proskauer test, hemolysis, and other tests. Morphological and biochemical characteristics including the colony characters of the ten isolates were not identical which proved that the structure and functions of the isolates were different. Of all the ten isolates, M3 and M8 (Fig. 1) were chosen out of interest and further selected for molecular characterization by 16S rRNA sequencing and phylogenetic analysis. The morphological and physiological characteristics of M3 and M8 were compared with the data from Bergey’s Manual of Determinative Bacteriology, and M3 was found to be a facultative anaerobe, motile, and gram-positive rod and M8 a facultative anaerobe, motile, and gram-negative rod.

**Table 2:** Biochemical characterization of isolated bacterial strain

S. No	Name of the Test	M1	M2	M3	M4	M5	M6	M7	M8	M9	M10
1.	Catalase activity	-	+	+	+	+	+	-	+	-	+
2.	Hemolysis	α	Α	β	γ	α	β	β	β	α	γ
3.	Oxidase	+	+	+	-	+	+	-	+	-	-
4.	Urease	+	-	-	-	-	-	-	-	-	-
5.	Methyl red	+	+	+	+	+	+	+	-	+	+
6.	Voges-Proskauer reaction	+	-	-	-	-	-	-	-	-	-
7.	Indole	-	-	-	-	-	-	-	-	-	-
8.	Starch hydrolysis	+	+	+	+	+	+	+	+	+	+
9.	Gelatin liquefaction	+	+	+	+	+	+	+	+	+	+
10.	Motility	M	NM	M	NM	M	NM	M	M	M	NM
11.	Nitrate reduction	+	_	+	+	+	+	-	+	-	+
12.	H_2_s production	+	+	+	+	+	+	+ -	-	+	+
13.	Citrate utilization	+	-	-	+	-	-	+	-	+	+
14.	Carbohydrate utilization test										
	Sucrose	A	A	A	A/G	A	A	A	A	A	A
	Maltose	A	A	A	A	A/G	A	A	-	A	A
	Glucose	A	A	-	A	A	A	A	A/G	A	A/G
	Lactose	A	-	A	A	A/G	-	A	-	A	A
	Mannitol	A	-	-	A	-	-	-	-	-	A
	Mannose	-	-	A	A	A	A	A		A	A

*+* positive, *–* negative, *M* motile, *NM* non-motile, *A* acid producer, *G* gas producer, *A/G* acid and gas producer

### Extraction of DNA, PCR amplification, and 16SrRNA gene sequencing of M3 and M8 isolates

**Fig. 2:**
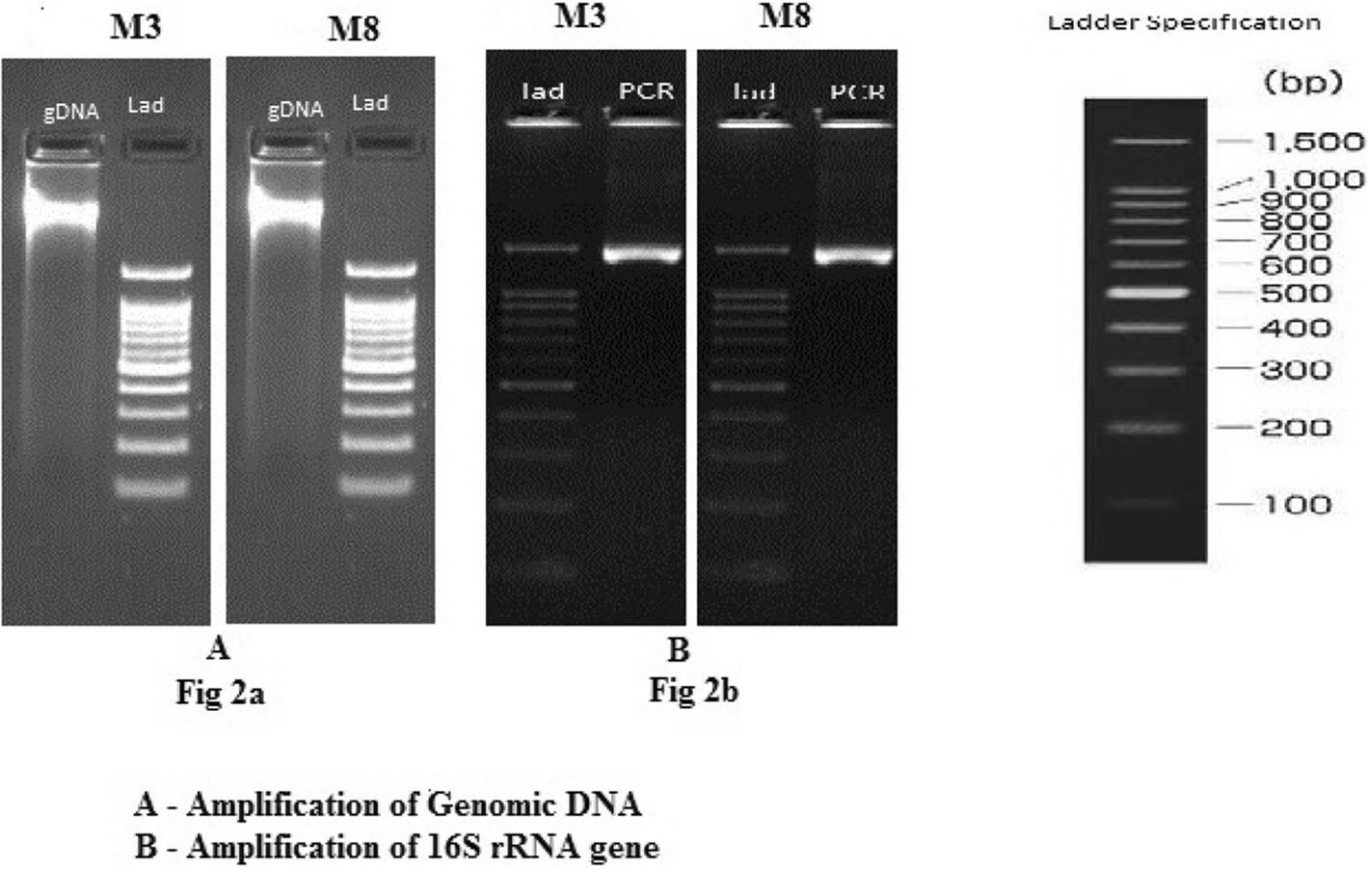
**A** Amplification of genomic DNA of M3 and M8 isolates. **B** Amplification of 16S rRNA gene of M3 and M8 isolates

DNA isolation, PCR amplification of 16SrRNA gene sequencing, and sequencing of rRNA fragment were done according to sarkosyl method [29]. DNA was isolated from the isolates, and the purity was evaluated on 1.0% agarose gel. A single band of high-molecular weight DNA has been observed (Fig. 2A).

A molecular approach was used to establish and support the identification of the isolates. The 16S rRNA gene has become a reliable tool for the identification and classification of bacteria. 16S rRNA gene studies are necessary to achieve unambiguous identification at the species level. Fragment of 16S rRNA gene was amplified by 27F and 1492R primers, and a single discrete PCR amplicon band of 1500 bp was observed when resolved on agarose gel for both the isolates (Fig. 2B). The PCR amplicon was purified to remove contaminants. Forward and reverse DNA sequencing reaction of PCR amplicon product was carried out with forward primer and reverse primers using BDT v3.1 Cycle sequencing kit on ABI 3730xl Genetic Analyzer. Consensus sequence of 16S rRNA gene was generated from forward and reverse sequence data using an aligner software. The 16S rRNA gene sequence was used to carry out BLAST with the database of NCBI Genbank. 16S rRNA sequences of both the isolates M3 and M8 were submitted to NCBI GenBank and got the accession numbers for both the isolates—M3 (*Bacillus subtilis* MZ047611) and M8 (*Pseudomonas resinovorans* MZ047610).

### Phylogenetic analysis

**Fig. 3:**
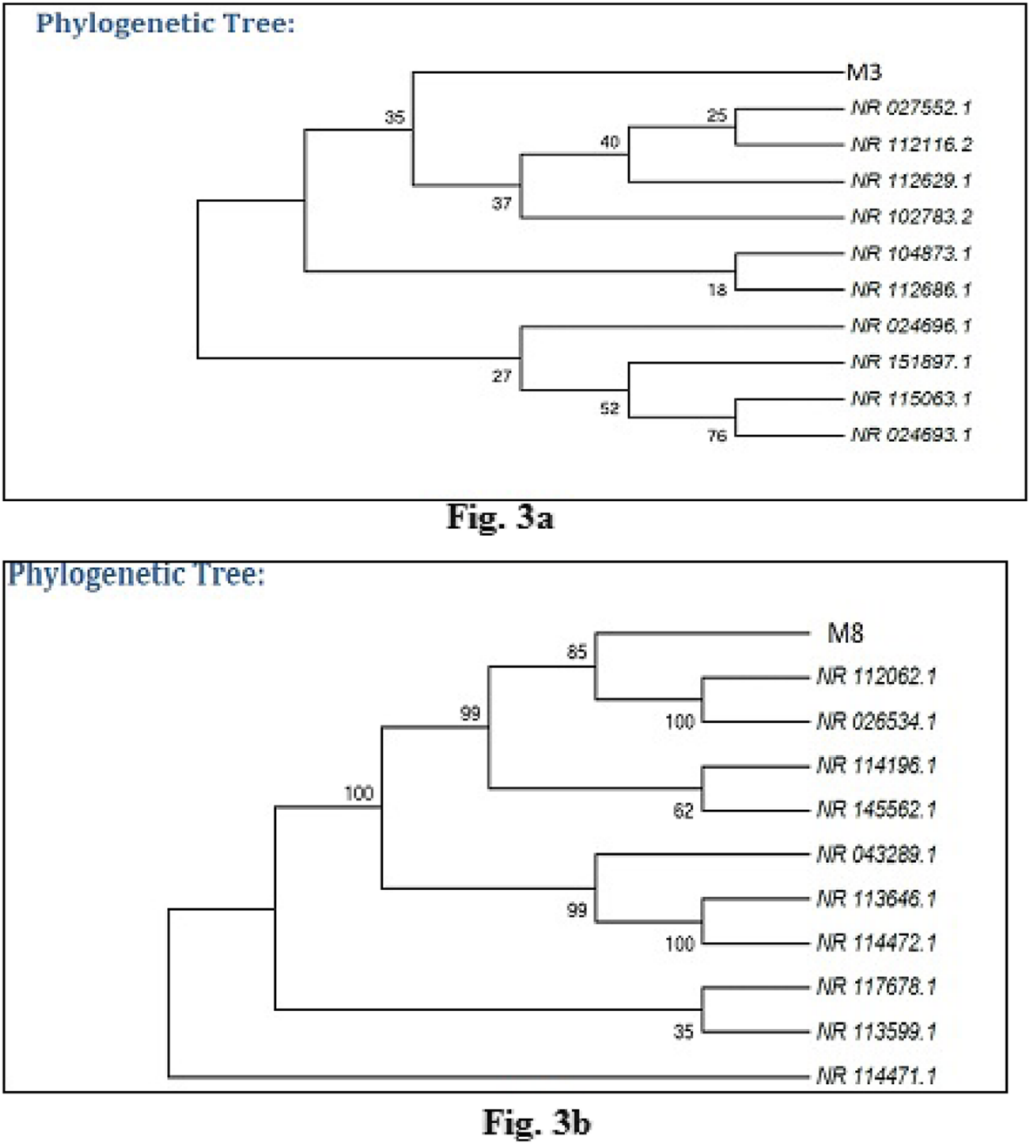
**a** Phylogenetic relationship of isolated strain M3. The tree is constructed using 16S rRNA sequence by maximum likelihood method. **b** Phylogenetic relationship of isolated strain M8. The tree is constructed using 16S rRNA sequence by maximum likelihood method

The 16S rRNA gene sequences of both the isolates were subjected to BLAST online tool in the database of NCBI Genbank. Phylogenetic analysis was performed based on maximum identity scores; first ten sequences were selected and aligned using multiple alignment software program Clustal W. The molecular and phylogenetic analysis of 16SrRNA gene sequences revealed the two isolates, M3 and M8, showed identity to *Bacillus subtilis* (98.24%) and *Pseudomonas resinovorans* (98.17%) respectively. Distance matrix was generated, and the phylogenetic tree was constructed using MEGA 7 (Fig. 3a and b).

The evolutionary history was inferred by using the maximum likelihood method based on the Kimura 2-parameter model [30]. Evolutionary analyses were conducted in MEGA7 [31]. The bootstrap consensus tree inferred from 1000 replicates is taken to represent the evolutionary history of the taxa analyzed [32].

### Applications of M3 and M8 isolates

#### Decolorization of dyes

**Fig. 4:**
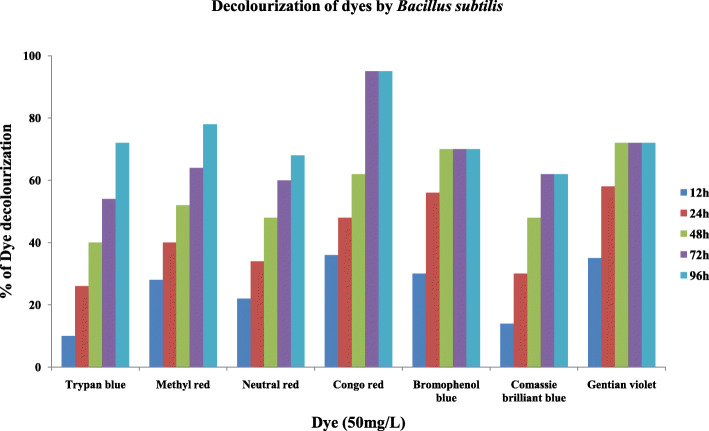
Decolorization of different dyes by *Bacillus subtilis* (M3)

**Fig. 5:**
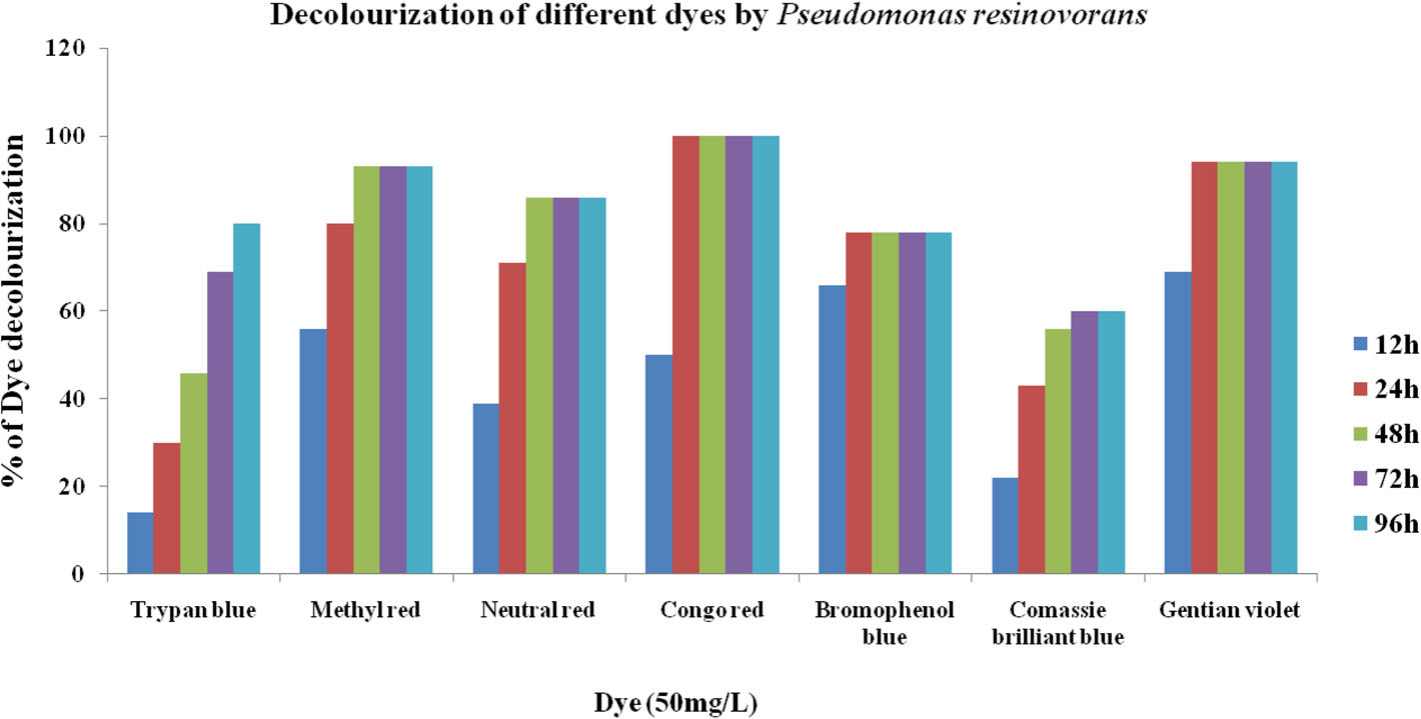
Decolorization of different dyes by *Pseudomonas resinovorans* (M8)

The isolates M3 (*Bacillus subtilis*) and M8 (*Pseudomonas resinovorans*) were tested for their ability to decolorize 50mg/L of seven dyes, namely, Trypan blue, Methyl red, Neutral red, Congo red, Bromophenol blue, Coomassie brilliant blue, and Gentian violet. The dye concentration was decreased with increase in period of time for all the dyes. The percent degradation of various dyes by the two isolates is shown in Figs. 4 and 5. Complete decolorization (100%) of the dye Congo red was achieved by the isolate M8, and the isolate M3 attained 95% of decolorization in 2 days of incubation. The isolate M3 decolorized Trypan blue, Methyl red, Neutral red, Bromophenol blue, Coomassie brilliant blue, and Gentian violet with 72% (96h), 78% (96h), 68% (96), 70% (48h), 62% (72), and 72% (48h) respectively. On the other hand, isolate M8 also decolorized Trypan blue, Methyl red, Neutral red, Bromophenol blue, Coomassie brilliant blue, and Gentian with 80% (96h), 93% (48h), 86% (48h), 78% (24h), 60% (72h), and 94% (24h) respectively.

#### Cadmium removal capacity of M3 and M8 isolates

**Fig. 6:**
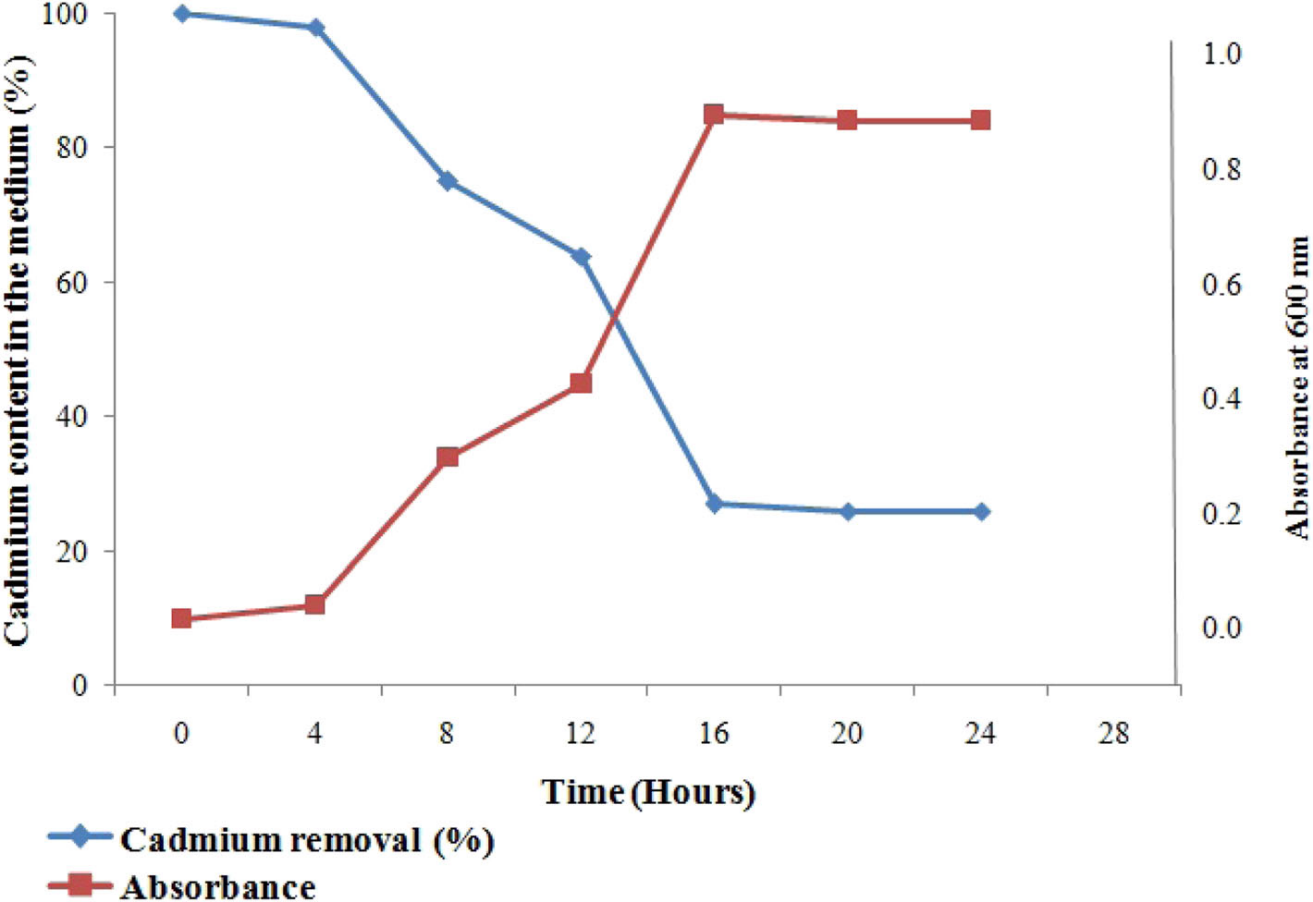
Removal of cadmium by *Bacillus subtilis* (M3)

**Fig. 7:**
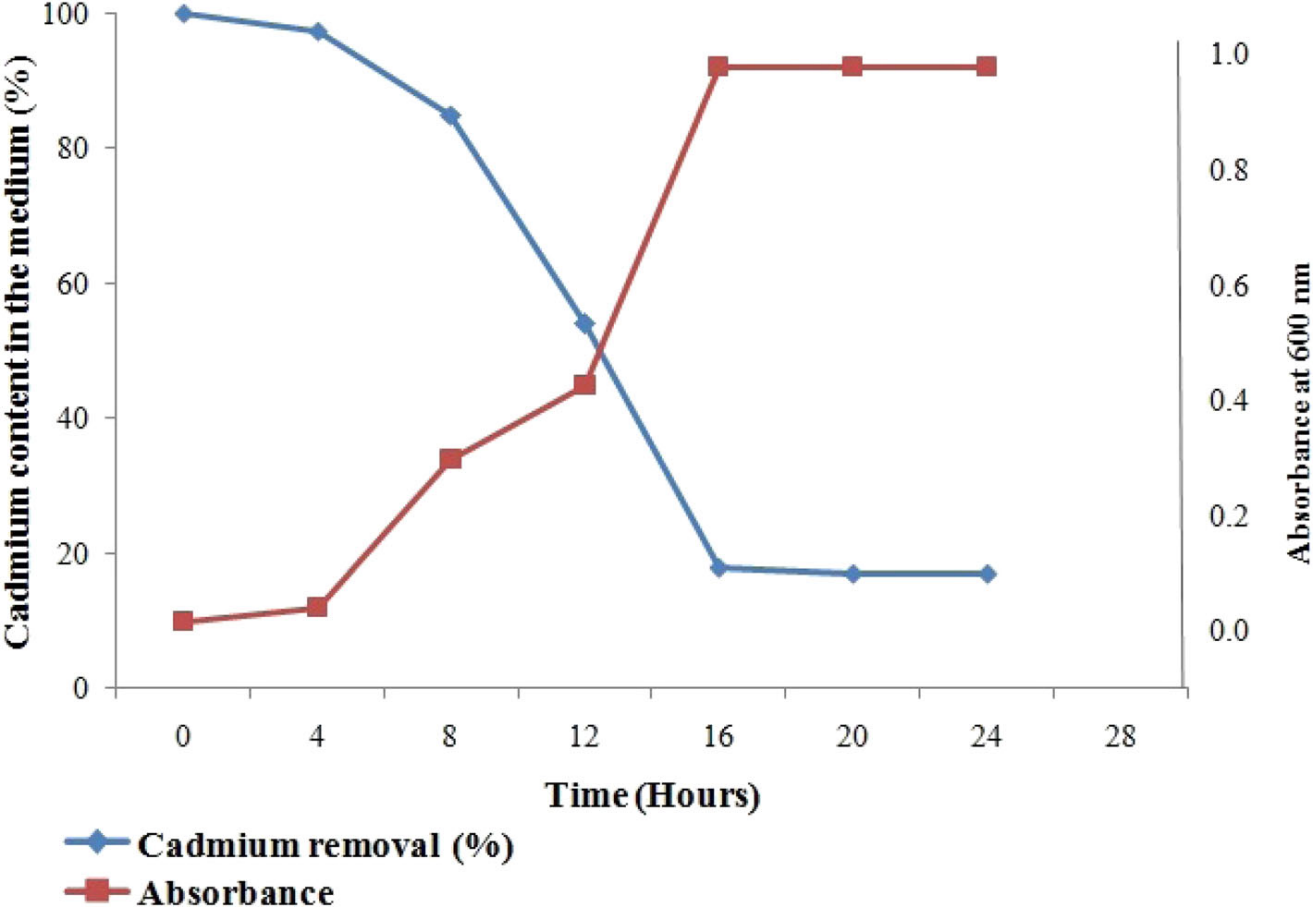
Removal of cadmium by *Pseudomonas resinovorans* (M8)

The cadmium removal capacity of the isolates M3 and M8 is shown in Figs. 6 and 7. The lag phase was observed during the initial 0–4 h which was another sign of the cadmium toxicity to both the isolates. In the lag phase, isolate M3 removed only 2.08% of the cadmium whereas the isolate M8 removed 4.32% of the cadmium from the medium. After that, in log phase (8–16 h), isolate M8 removed the maximum amount of the cadmium which is about 84.70%, and the isolate M3 removed 74.14% because in this phase bacteria rapidly divide with time, so bioaccumulation of the cadmium also increased. The percentage of the cadmium bioaccumulation by both isolates M3 and M8 was increasing from the beginning up to 16 h. After 16 h, a slight reduction in cadmium removal was observed from the medium at stationary phase for both the isolates.

## Discussion

Marine microorganisms have developed unique metabolic and physiological capabilities that not only ensure survival in extreme habitats but also offer the potential for the production of metabolites [33, 34]. Marine microorganisms have significant osmotic tolerance leading to their capability to survive at higher salt concentration [35]. Microorganisms are considered to be the best indicators of changes in environmental conditions and are sensitive to low concentration of heavy metals but rapidly adapt to the specific habitat conditions [36]. Nowadays, bioremediation has become one of the promising approaches for the removal of toxic textile synthetic dyes which were expelled from textile, paper, printing, and mineral processing industries. It is a well-known fact that several microorganisms including bacteria, fungi, yeasts, and algae can completely decolorize many dyes [37].

In the present study, ten bacterial strains were isolated from marine water collected from Visakhapatnam harbor by serial tube dilution method. All the isolates showed different morphological and colonial characteristics on nutrient agar medium. Biochemical characterization was done for all the isolates, and they differed in their biochemical characteristic properties. The morphological and biochemical characteristics of M3 and M8 were compared with the data from Bergey’s Manual of Determinative Bacteriology, and M3 was found to be a facultative anaerobe, motile, and gram-positive rod and M8 a facultative anaerobe, motile, and gram-negative rod. Isolates M3 and M8 were selected for molecular characterization by 16S rRNA sequencing and phylogenetic analysis. Molecular characterization by 16S rRNA gene sequence analysis is a cost-effective tool to identify simpler to complex microbial strains at species level. The phylogenetic analysis was performed for both the isolates using 16S rRNA gene sequences to identify microbial strains at species level. The amplified gene sequences were aligned with closely related strain sequences retrieved from NCBI database obtained by BLAST search. The molecular characterization and phylogenetic analysis of the gene sequences by maximum likelihood method revealed that the two isolates, M3 and M8, showed identity to *Bacillus subtilis* (98.24%) and *Pseudomonas resinovorans* (98.17%) respectively. Ivanova et al. [38] reported taxonomically characterized *Bacillus* strains isolated from marine waters of the Pacific Ocean. Several *Bacillus sp.* have been reported from marine environment by molecular characterization of 16S rRNA gene sequence [39, 40]. 16S rRNA gene sequencing was also allowed in the identification in the several *Pseudomonas sp.* [41].

The isolates M3 and M8 effectively decolorized 50mg/L of seven textile dyes, namely, Trypan blue, Methyl red, Neutral red, Congo red, Bromophenol blue, Coomassie brilliant blue, and Gentian violet. Interestingly, M3 has completely (100%) decolorized Congo red whereas M8 decolorized 95% of the dye. Sharma et al. [42] reported high dye decolorization ability of *Bacillus sp.*, *Alcaligenes sp.*, and *Aeromonas sp*., isolated from soil and sludge samples.

The decolorizing *Bacillus* and *Pseudomomas* isolates were also reported from the textile effluent samples [43]. Kumar and Sawhnei [44] reported that *Bacillus subtilis*–RA29 efficiently degraded Congo red (95.6%) at 37°C. Microorganisms can be used to completely degrade the dyes because they reduce the dyes by secreting enzymes such as laccase, azoreductase, peroxidase, and hydrogenase [45]. Decolorization of dyes may take place either by adsorption onto the microbial biomass or biodegradation of dyes by the cells [46]. The possibility of using novel microbial consortium for biological treatment of disreputable dyeing effluents has been reported by Karim et al. [47].

The natural marine bacterial isolates have got tremendous potential to tolerate, sequester, and remove toxic metal pollutants from the ambient environment. Therefore, the present study focused to check the capability of marine bacterial isolates M3 and M8 with reference to their role in the removal of cadmium from the growth medium. The M8 effectively removed the maximum amount of cadmium from the growth medium to about 84.70%, and M3 removed 74.14% of cadmium from the growth medium. The findings of the study revealed that both the isolates can be recommended in the bioremediation of cadmium from the pollutant environment.

Heavy metal-resistant microorganisms play an important role in the bioremediation of heavy metal-contaminated environment [48, 49]. Response to varying concentrations of cadmium on *Bacillus* sp. isolated from Indian coastal waters was reported by Nair et al. [50]. Several studies have reported the ability of gram-negative bacteria to resist and accumulate cadmium ions [51]. *Pseudomonas aeruginosa* biomass was reported and effective bio adsorbent for the removal and recovery of cadmium from polluted water [52].

## Conclusions

The current study has been focused on the isolation and characterization of bacterial isolates, M3 and M8 from the marine water of Visakhapatnam harbor. The isolates were characterized by using biochemical test and 16S rRNA molecular sequencing followed by phylogenetic analysis. The results of molecular characterization revealed that the isolates M3 have 98.24% identity to *Bacillus subtilis* and M8 have 98.17% towards *Pseudomonas resinovorans*. The isolates effectively decolorized textile dyes and removed cadmium from the growth medium. Overall results in the present suggest that isolates M3 and M8 could be used in the bioremediation of heavy metals from the pollutant environment and also in the degradation of textile dyes. Further studies are needed to support the experimental work in studying the molecular mechanism of dye decolorization by the isolates M3 and M8. The eco-friendly bioremediation of cadmium from the medium by M3 and M8 isolates offer a great choice to eliminate toxic cadmium permanently from the environment and consequently supports their potential as bioremediation agents in polluted environments indicating the potential of isolates as biosorbent for removal of high concentration of heavy metals from wastewater and industrial effluents.

## Supplementary Information

**Additional file 1.** Supplementary file 1
